# Challenges encountered in executing family routines: a comparison between neurotypical children and those having attention deficit hyperactivity disorder or autism spectrum disorder

**DOI:** 10.3389/frcha.2026.1758537

**Published:** 2026-03-17

**Authors:** Rosalie Ruel, Line Massé, Dany Lussier-Desrochers, Claudia Verret, Isabelle Simonato

**Affiliations:** 1 Department of Psychoeducation, Université du Québec à Trois-Rivières, Trois-Rivières, QC, Canada; 2 Department of Physical Activity Sciences, Université du Québec à Montréal, Montréal, QC, Canada; 3 University Institute of Intellectual Disability and Autism Spectrum Disorder, Trois-Rivières, QC, Canada

**Keywords:** attention-deficit/hyperactivity disorder, autism spectrum disorder, children, family, routine, neurodevelopmental disorders

## Abstract

**Introduction:**

Daily routines play a central role in the child's development process and the establishment of harmonious family dynamics. However, many parents of children with attention-deficit/hyperactivity disorder (ADHD) and with autism spectrum disorder (ASD), report difficulties in establishing and maintaining routines. The aim of this study was to compare neurotypical, ASD, and ADHD children's performance on daily routines. Precisely, it aims to describe the difficulties, the impacts on the child and his family, and the nature of the difficulties.

**Method:**

The sample consisted of 205 children aged 6 to 12 years (*M* = 8.60, *SD* = 1.78; 31.7% girls), including 104 children with ADHD, 49 children with ASD, and 52 neurotypical children. Analyses of covariance (ANCOVAs) were performed to compare the three groups of participants, controlling for children's age and gender, parental education and family structure.

**Results:**

The results show that families of children with ADHD generally perceive routines as more difficult than those of neurotypical children. Children with ADHD experience significantly more frustration than neurotypical children when performing routines. According to parents, these difficulties in carrying out daily routines adversely affect the family climate, making it more stressful and unpleasant.

**Discussion:**

In conclusion, understanding the differences in the difficulties faced by these three groups of children in carrying out their daily routines will make it easier to support families in implementing interventions that are better adapted to the child's specific needs.

## 1 Introduction

Researchers have extensively studied routine performance, primarily because they play a crucial role in the daily lives of families. Routines foster family unity by providing a defined structure for the activities that parents and children must complete (1–5). The successful implementation of routines is associated with several benefits for both children and parents (6). Routines foster positive interactions between parents and children, thereby enhancing family cohesion by providing structure (3–5). Thus, the family experiences a positive and enjoyable environment, which subsequently enhances the overall well-being and quality of life of all individuals involved (7–11). Integrating children into the daily family routine offers them multiple opportunities for varying learning and fosters the development of their sense of responsibility and independence, which are critical requirements for success as future members of society (4, 9, 12, 13). Furthermore, research links living in a consistent, organized, and unambiguous environment to a considerable array of advantages for both children and their parents (6). Creating and sticking to clear routines can help lower the risk of both internalized and externalized behaviour issues, deal with common discipline problems related to getting ready for school, doing homework, and going to bed, and it can improve academic performance (9, 14–20). In addition, routines promote children's autonomy and self-esteem, along with enhanced well-being and executive functions (12, 21–23). Consistent and efficient routines increase parents's satisfaction and reduce stress levels, creating a more enjoyable and supportive family environment that fosters the development of meaningful shared experiences (24, 25).

A routine is a systematic and foreseeable sequence of activities defined by established guidelines and executed daily or weekly (26, 27). It relates to events that occur in daily life (such as waking, dining, and sleeping). It involves at least two family members (25). The varied routines that shape family life promote the achievement of goals, the structuring of family time, and the strengthening of connections among family members (28, 29). Furthermore, routines provide children with a distinct understanding of the expectations their parents have for them (2, 27). According to Barkley (30), a routine should be adjusted according to the time of day (morning, evening, hygiene), the time of year (school period or holidays), and the age of the children.

Families with a child who has special educational requirements or who expresses particular behaviours such as resistance, stereotypies, or sensory needs, may face additional challenges in their daily routine, which can have a significant impact on the family unit. Research indicates that numerous families of children diagnosed with attention-deficit/hyperactivity disorder (ADHD) or autism spectrum disorder (ASD) experience more challenges in executing their everyday activities (1, 5, 14, 31, 32). Although parents may have strong intentions, they may face challenges due to fatigue, lack of motivation, or even a deficiency in solutions (3, 19). The implementation of a routine requires substantial time, consistent practice, and unwavering dedication. Therefore, the problematic or particular behaviours exhibited by a child (e.g., resistance, inattention, stereotypies, sensory abnormalities) can disrupt routines and provide an additional obstacle (19, 33).

Based on Barkley (34) and Sinzig et al. (35), the challenges faced in carrying out daily tasks are typically associated with the primary features or symptoms of ASD or ADHD. Briefly, according to the Diagnostic and Statistical Manual of Mental Disorders-5-Text Revision (DSM-5-TR; 36), neurodevelopmental disorders, which include ADHD, generally appear in early childhood. They are characterized by developmental delays that can affect a variety of skills, including behavioural, sensorimotor, and cognitive skills. Representing a persistent pattern of inattention and/or hyperactivity-impulsivity, ADHD symptoms must significantly impair the activities of daily living (36). In Canada, the prevalence of this disorder is estimated at 11.3% for people aged between 1 and 24, with a ratio of 2 boys to 1 girl (37). ASD is characterized by persistent deficits in social communication and social interactions across multiple contexts. Also, the person's behaviours, interests, or activities are characterized as restricted and repetitive (36). According to Statistics Canada [2019, cited in (38)], the prevalence of ASD is approximately 2.0% among Canadian youth aged 1 to 17.

The acquisition, consolidation and execution of routines pose significant challenges for children diagnosed with ASD or ADHD, as they may exhibit impairments in their executive functioning (13, 39). Executive functioning is a multifaceted cognitive concept that refers to a set of operations that control and govern an individual's activities to accomplish a future goal (40, 41).

In this regard, several papers have compared the executive functions of people with ASD with those of people with ADHD or neurotypicals in order to identify differences or similarities in their profiles. According to Townes et al. (42), the executive functioning of people with ASD or ADHD differs significantly from that of neurotypicals, notably by a more marked presence of deficits in inhibition, cognitive flexibility, attention, and visuospatial skills. However, there were no differences in working memory or speed of information assimilation. These research findings are corroborated by other authors (43, 45). However, comparing the executive functioning of people with ASD with that of people with ADHD yields different results. Indeed, Corbett et al. (44) report that people with ASD have more difficulty with inhibition, working memory and cognitive flexibility than people with ADHD. However, according to Townes et al. (42), there appear to be no significant differences between these two groups in terms of executive functioning. Ceruti et al. (45) add, however, that the data collection instrument used to attest to the executive functioning of people with ASD and those with ADHD greatly influences the results obtained. Indeed, when assessed using a neuropsychological test, the executive functioning of these two groups does not differ significantly. Conversely, when questionnaires are used, the results highlight a significant difference between people with ASD and those with ADHD in terms of executive functions (45). In short, as highlighted in the review by Liu et al. (43), studies report as many similarities as differences in deficits in executive functioning between people with ASD and those with ADHD.

Yet, although establishing routines is more challenging for families with children with ASD or ADHD, several studies report that carrying out routines on a daily basis is strongly associated with lower levels of children's internalized and externalized behaviours (18, 46–48). The predictability associated with routines helps children understand their responsibilities and expectations, potentially reducing the inattention or stress associated with their disorder's characteristics (18, 20).

When a child has difficulty performing routines, this has repercussions for all family members, affecting overall family functioning (33, 49, 50). Parents of children having ASD in the study by Schaaf et al. et al. (32) identified the morning routine as particularly strenuous for their children, citing time constraints such as leaving for school and school transport. Indeed, parents were preoccupied with their children's routines, while they in turn had to get ready for their personal occupations (e.g., work). In a qualitative study of 14 families with children having ASD aged 2 to 5 (33), 92.0% of mothers reported that the mealtime routine was the most stressful for them. Considering their child's sensory sensitivities (e.g., olfactory, gustatory), mothers must constantly adjust to these particularities, which considerably reduced the attention paid to other family members. Also, several mothers in the Marquenie et al. (33) study mentioned that, unlike the mealtime routine, the bedtime routine offers a more predictable framework for their child. Although the latter also represents a challenge for parents, they reported that they can share more quality time with their child before bedtime. According to Shikerkar and Vajaratkar's (5) study of 10 parents of children with ADHD, the majority reported having difficulties managing family routines because their child depended heavily on the verbal cues offered by the parent to carry out the sequence. Furthermore, in a qualitative study of 17 families of children with ADHD aged 6 to 17 (51), 52.9% of parents mentioned that the routine following the return from school (often related to homework) was the most difficult to carry out, followed by the morning routine. Parents described these routines as “chaotic” and “stressful” (51). In the same vein, the morning routine would be associated with a greater intensity of ADHD symptoms, impacting the tasks to be accomplished and generating negative emotions for the whole family (50). In sum, for both parents of children with ASD or with ADHD, the daily routines seem to present a significant challenge, mobilizing a tremendous deal of energy and time for the family. However, the challenges encountered and difficulties reported seem to differ somewhat between children in the two groups. Despite that, parents of children with ASD or ADHD commonly expressed that establishing and maintaining routines is a major source of exhaustion, overload, and stress for them (32, 50, 52–54). Parents may be more likely to berate their child and make negative remarks by being more tired, more stressed, and less rested, thereby reducing positive family interactions (55).

Few studies looked at the day-to-day lives of families of neurotypical children and children with ASD or ADHD. Although the parents of these children commonly report difficulties with daily routines, the challenges encountered may be actualized differently, particularly in relation to executive functioning. Consequently, current research does not allow us to understand how children's specific characteristics impact routine execution, the conditions that support or hinder routine implementation, or the daily outcomes for these families. In order to facilitate the implementation of adapted interventions to help children carry out their routines, it's critical to understand how the difficulties occur precisely in these three groups of children (ASD, ADHD, and neurotypical). To achieve this, it is important to compare whether there are similarities or differences in the difficulties experienced.

The aim of the study is to compare neurotypical children with children having ASD or ADHD on various aspects related to the performance of daily routines, including (1) the routines most difficult to perform; (2) the impact of the performance of routines on the child; (3) the impact of the performance of routines on family life; and (4) the nature of the difficulties.

## 2 Method

A quantitative comparative design was used to examine the similarities and differences between groups (56), considering the presence or absence of a neurodevelopmental disorder (ASD or ADHD).

### 2.1 Participants

The present research is part of a larger study evaluating the effects of a digital application on the performance of routines by neurotypical children and children with ASD or ADHD aged 6 to 12 (65). This study was approved by the research ethics committee of the Université du Québec à Trois-Rivières (CER-19-261.07.03). This study was held in partnership with the Neuro Solution Group, a partner who has actively contributed to the study with the database management.

Participants for this larger study were recruited using a non-probability sampling method. Families were first invited to participate by giving their information on the website of the project. The research team contacted 320 of the 383 families who agreed to share their information, as they met the project's inclusion criteria. These were: (1) having a child aged 6 to 12; (2) living in Quebec; (3) having a confirmed diagnosis of ADHD, ASD, or no diagnosis for the child; and (4) having only one child in the family to participate in the study. As the broader study involved the use of a digital application, the target child was expected to demonstrate adequate ability to use it.

For the current article, data were extracted from the principal database developed with the 205 French-speaking families that gave their consent to take part in the study. The mother was the primary parent responding to the questionnaires (93.6%). Fathers were aged between 28 and 70 (*M* = 40.27 years; *SD* = 5.69) and mothers between 28 and 62 (*M* = 37.97 years; *SD* = 5.06). At least one parent in 55.7% of families held a university degree, while 14.7% of families consisted of a single child.

The study targeted only one child per family, with the parent respondent's responses focusing on this child. Of the 205 children targeted, 65 (31.7%) were girls and 140 (68.3%) were boys. The children were aged between 6 and 12, with an average age of 8.60 years (*SD* = 1.78 years). Neurotypical children were significantly younger (*p* < .05; *M* = 7.67 years; *SD* = 1.54 years) than those with a diagnosis of ADHD (*M* = 8.94 years; *SD* = 1.72 years) or ASD (*M* = 8.86 years; *SD* = 1.84 years). The majority of the sample (50.7%) included children with ADHD. Children with ASD represented 23.9% of the sample, while neurotypical children accounted for 25.4%. Table 1 presents the characteristics of the children.

**Table 1:** Characteristics of children in the three groups (*n* = 205).

Characteristics	Neurotypical children	Children with ADHD	Children with ASD	Total
	*n* (%)	*n* (%)	*n* (%)	*n* (%)
Girl	27 (51.92)	29 (27.88)	9 (18.37)	65 (31.71)
Boy	25 (48.08)	75 (72.12)	40 (81.63)	140 (68.29)
	*M (SD)*	*M (SD)*	*M (SD)*	*M (SD)*
Age	7.67 (1.54)	8.94 (1.72)	8.86 (1.84)	8.60 (1.78)

### 2.2 Measurement procedure and instruments

Only children with a diagnosis of ASD or ADHD confirmed by a qualified professional could take part in the study. As the participants were not asked to provide any proof of their diagnosis, the research team e-mailed the French version of the Behavior Assessment System for Children 3—Parent Version (BASC-3; 57) questionnaire to the responding parent to confirm the children's diagnosis once recruitment has been completed Subsequently, the research team sent the electronic version of the sociodemographic questionnaire, the questionnaire on challenges encountered, and the routine performance questionnaire to the responding parent.

#### 2.2.1 Sociodemographic questionnaire

The sociodemographic questionnaire gathers information about the family's characteristics. The questionnaire inquired about the parents' age, gender, place of residence, and the highest level of education they had completed. It also inquired about the family's composition, including its type and the number of children living within it. Lastly, the questionnaire asked the child's age, gender, and whether they had ADHD, ASD, or no diagnosis. The sociodemographic questionnaire takes about 5 min to complete.

#### 2.2.2 Questionnaire on difficulties encountered during home routines

The research team designed the Questionnaire on Difficulties Encountered During Home Routines to understand how the targeted child implements routines at home. Researchers first conducted a review of the literature on the challenges children face during routines to create this questionnaire. Then, as part of another project, the research team conducted a needs study with parents, and the results allowed to adjust the questionnaire to its current version. Specifically, the questionnaire comprises 23 items divided into four main themes: difficulties with daily routines (5 items; α = .77), the impact of routines on the child (8 items; α = .76), and the impact of routines on family life (10 items; α = .81). Participants must indicate their level of agreement on a 6-point Likert scale ranging from strongly disagree (1) to strongly agree (6). This questionnaire takes between 5 and 10 min to complete.

#### 2.2.3 Routine performance questionnaire

Simonato et al. (58) developed and validated the Routine Performance Questionnaire (RPQ). It assesses the types of challenges faced through observable behaviors, potentially impacted by specific executive functions. Specifically, the questionnaire comprises 19 items divided into four scales: (1) working memory (4 items); (2) behavioural self-regulation (7 items); (3) planning and time management (6 items); and (4) emotional self-regulation (2 items). This instrument uses a 6-point Likert scale ranging from strongly disagree (1) to strongly agree (6) and takes between 5 and 10 min to complete. The RPQ provides a score for each of the four scales, as well as an overall score, which provides information about the nature of the child's difficulties. According to the properties of this questionnaire, the lower the score, the more difficulties the child has with behaviours associated with performing routines. Conversely, the higher the score, the more easily the child performs routines. This questionnaire presents good internal consistency indices (ω = .71 to. 85; 58).

### 2.3 Analysis

First, descriptive analyses were performed on the participants' sociodemographic data and on the difficulties encountered during home routines and the RPQ. Analysis of covariance (ANCOVA) were performed on difficulties encountered and RPQ for determining whether there were statistically significant differences between independent groups, after controlling for certain variables (59). The Bonferroni *post-hoc* test was used to assess differences between groups (*p* < .05). Four variables were controlled for the ANCOVA: children's age and gender (1 = boy), parental education (1 = university degree) and family structure (1 = single parent family). To maximize the data collected, the analyses were conducted on a sample size ranging from 202 to 172 participants. There were two main reasons for this variation: (1) participants had the option of choosing “does not apply” in the questionnaire of difficulties encountered in home routines and the RPQ; and (2) some participants failed to answer a few questions. A maximum of two missing answers per participant was tolerated. IBM SPSS software (version 29.0) was used for analyses.

## 3 Results

The results obtained are presented in four sections: (1) difficulties with daily routines; (2) the impact of performing routines on the child; (3) the impact of performing routines on family functioning; and (4) the nature of the difficulties.

### 3.1 Difficulties with daily routines

Table 2 presents the results of descriptive analyses and ANCOVA. Descriptive results show that the meal period was the easiest routine for all three groups, while the other routines seemed more difficult. ANCOVA results show that children with ADHD had significantly more difficulty performing the morning routine compared to neurotypical children (*p* < .001) and to children with ASD (*p* < .01) with a medium effect size (partial *η*² = .10). Also, children with ADHD had significantly more difficulties than neurotypical children when completing homework (*p* < .05). The effect size (partial *η*^2^) was .04, indicating a small effect size. No statistically significant differences were found regarding mealtime and evening routines between the three groups.

**Table 2:** Results of descriptive analyses and ANCOVA on the difficulties encountered with daily routines.

Routines	1-Neurotypical children	2-Children with ADHD	3-Children with ASD	Total	ANCOVA			Post hoc Bonferroni
	Mean (*SD*)	Mean (*SD*)	Mean (*SD*)	*n*	*F*	df	*p*	
Morning	4.08 (1.38)	4.96 (1.03)	4.19 (1.33)	200	10.780	2	<.001^a^	2 > 1*** 2 > 3**
Meals	3.72 (1.55)	3.93 (1.44)	3.79 (1.35)	195	0.430	2	.651	–
Homework	4.19 (1.42)	4.97 (1.22)	4.82 (1.48)	185	3.698	2	.027	2 > 1*
Evening	4.41 (1.20)	4.68 (1.06)	4.30 (1.47)	201	1.857	2	.159	–

Control variables for ANCOVA: age and gender of children (1 = boy), parental education (1 = university degree) and family structure (1 = single parent family).

^a^ Levene's test of equality of variances significant at *p* < .05.

* *p* < .05.

** *p* < .01.

*** *p* < .001.

### 3.2 Impact of routines on the child

Table 3 presents descriptives and ANCOVA analyses on parents' perceived impact of performing routines on their child. According to descriptive results, frustration was the most common impact for all three groups of children, while delays at school were the least common. According to the ANCOVA results, children with ADHD show significantly more frustration than neurotypical children (*p* < .05). The effect size (partial *η*²) was.04, indicating a small effect size. Furthermore, neurotypical children received significantly fewer reprimands than ADHD children when they had difficulty performing a routine (*p* < .05), this effect being small (partial *η*² = .04). Also, the results reveal no significant differences between the impact reported for the three groups of children in terms of fatigue, stress, crying, school delays, and negative consequences, meaning that the difficulties encountered are similar. Finally, a mean score for the impact of routine performance on the child was calculated (7 items). For this total impact scale, Cronbach's alpha was .76. The results for the total score indicate that children with ADHD react more strongly than neurotypical children when they encounter difficulties (*p* < .05) in the execution of their routine, although the effect size is small (partial *η*² = .04).

**Table 3:** Results of descriptive analyses and ANCOVA on the impact of routines on the child.

Impacts	1-Neurotypical children	2-Children with ADHD	3-Children with ASD	Total	ANCOVA			Post hoc bonferroni
	Mean (*SD*)	Mean (*SD*)	Mean (*SD*)	*n*	*F*	df	*p*	
Is tired	3.45 (1.49)	3.75 (1.40)	4.17 (1.42)	197	2.840	2	.061	–
Is stressed	3.62 (1.35)	4.26 (1.46)	4.34 (1.54)	201	2.823	2	.062	–
Experiences frustration	4.38 (1.42)	5.05 (1.04)	4.70 (1.46)	202	4.424	2	.013^a^	2 > 1*
Cry	3.15 (1.58)	3.48 (1.59)	3.28 (1.66)	199	1.189	2	.307	–
Delays at school	2.46 (1.70)	2.97 (1.59)	3.07 (1.58)	193	1.280	2	.281	–
Receives reprimands	3.92 (1.51)	4.52 (1.24)	4.13 (1.55)	200	3.456	2	.034	2 > 1*
Receives negative consequences	3.81 (1.41)	4.02 (1.44)	3.58 (1.51)	199	1.523	2	.221	–
Total impact	3.56 (0.92)	4.02 (0.83)	3.91 (1.12)	202	3.717	2	.026^a^	2 > 1*

Control variables for ANCOVA: age and gender of children (1 = boy), parental education (1 = university degree) and family structure (1 = single parent family).

^a^ Levene's test of equality of variances significant at *p* < .05.

* *p* < .05.

### 3.3 Impact of routines on family life

Table 4 presents the results of descriptive analyses and ANCOVA on the impact of routine performance on family life. Descriptive results show that the main impact of difficulties with routines is the repetition of instructions by parents of all three groups of children. However, these difficulties with routines rarely caused parents in the three groups to be late for work. ANCOVA results showed that parents of children with ADHD reported having to repeat instructions more than parents of children with ASD (*p* < .05). The effect size (partial *η*²) was.04, indicating a small effect size. Also, parents of children with ADHD required significantly more continuous supervision of their child (*p* < .01; partial *η*² = .05), reported more impatience (*p* < .01; partial *η*² = .05), and were more frequently late for work (*p* < .01; partial *η*² = .06) than parents of neurotypical children. Furthermore, in families of a child with ADHD, the climate during the performance of routines was significantly more stressful (*p* < .01; partial *η*² = .05) and unpleasant (*p* < .05; partial *η*² = .04) compared to families with a neurotypical child. No statistically significant differences were observed between the three groups of children regarding anger, arguments between parents, and arguments between siblings. Finally, to construct a mean score for the impact of routine performance on family life, the nine repercussions listed in Table 4 were grouped together. Cronbach's alpha for this scale is.81. The mean score indicates that in families with a child with ADHD, family life was significantly more affected than in those with a neurotypical child (*p* < .01). The effect size (partial *η*^2^) was .07, indicating a medium-sized effect.

**Table 4:** Results of descriptive analyses and ANCOVA on the impact of routines on family life.

Impacts	1-Neurotypical children	2-Children with ADHD	3- Children with ASD	Total	ANCOVA			Post hoc bonferroni
	Mean (*SD*)	Mean (*SD*)	Mean (*SD*)	*n*	*F*	df	*p*	
Repeat instructions	5.42 (1.14)	5.78 (0.63)	5.40 (1.14)	202	4.456	2	.013^a^	2 > 3*
Constant supervision	4.92 (1.10)	5.44 (0.81)	5.23 (1.15)	202	5.250	2	.006	2 > 1**
Being impatient	4.44 (1.23)	5.04 (0.95)	4.70 (1.30)	202	4.719	2	.010	2 > 1**
Being angry	3.81 (1.52)	4.32 (1.24)	3.98 (1.19)	200	2.994	2	.052	–
Arguments between parents	3.31 (1.72)	3.60 (1.61)	3.05 (1.34)	172	1.706	2	.185	–
Arguments between siblings	3.98 (1.59)	4.40 (1.25)	4.12 (1.21)	181	1.752	2	.177	–
A stressful family climate	3.81 (1.62)	4.62 (1.24)	4.36 (1.31)	202	4.790	2	.009^a^	2 > 1**
An unpleasant family climate	3.50 (1.71)	4.19 (1.41)	3.83 (1.46)	202	3.674	2	.027^a^	2 > 1*
Late for work	2.27 (1.62)	3.15 (1.69)	2.82 (1.67)	180	5.061	2	.007	2 > 1**
Total impact	3.97 (0.94)	4.54 (0.72)	4.22 (0.97)	202	7.743	2	.001	2 > 1**

Control variables for ANCOVA: age and gender of children (1 = boy), parental education (1 = university degree) and family structure (1 = single parent family).

^a^ Levene's test of equality of variances significant at *p* < .05.

* *p* < .05.

** *p* < .01.

### 3.4 The nature of the difficulties encountered

The nature of the difficulties encountered was investigated using the RPQ (see Table 5). Descriptive results show that for all three groups, behaviors related to self-regulation were more difficult, while those related to working memory and emotional self-regulation were generally easier. According to the ANCOVA results, children with ASD have significantly greater facility with behavioral self-regulation than their peers with ADHD (*p* < .05). For example, children with ADHD were more often distracted and delayed in initiating their routines. The effect size (partial *η*²) is.04, indicating a small effect size. In addition, planning and time management appear to be significantly easier in neurotypical children than in children with ADHD (*p* < .05; partial *η*² = .05). The results on the global score generated on the RPQ indicate that neurotypical children have significantly less difficulty in carrying out their daily routines than children with ADHD (*p* < .05), but the effect size was small (partial *η*² = .05). The results show no statistically significant differences between the three groups of children with regard to behaviours related to working memory and emotional self-regulation.

**Table 5:** Descriptive analyses and ANCOVA results concerning the nature of difficulties encountered.

Nature of difficulties	1-Neurotypical children	2-Children with ADHD	3- Children with ASD	Total	ANCOVA			Post hoc bonferroni
	Mean (*SD*)	Mean (*SD*)	Mean (*SD*)	*n*	*F*	df	*p*	
Working memory	3.52 (0.90)	3.19 (0.94)	3.38 (0.83)	202	1.555	2	.214	–
Behavioral self-regulation	2.41 (0.92)	1.98 (0.67)	2.33 (1.13)	202	4.147	2	.017^a^	3 > 2*
Planning and time management	2.82 (0.96)	2.36 (0.85)	2.50 (0.89)	202	3.261	2	.040	1 > 2*
Emotional self-regulation	3.37 (1.11)	3.19 (1.15)	3.07 (1.05)	202	0.928	2	.397	–
Total	2.87 (0.61)	2.49 (0.56)	2.69 (0.77)	202	4.984	2	.008^a^	1 > 2*

Control variables for ANCOVA: age and gender of children (1 = boy), parental education (1 = university degree) and family structure (1 = single parent family).

a Levene's test of equality of variances significant at *p* < .05.

* *p* < .05.

## 4 Discussion

The goal of this study was to compare the performance of daily routines by neurotypical children with ASD or ADHD from their parents' point of view. This study highlighted distinctions and similarities in the difficulties encountered by the three groups of children in carrying out their routines. It also allowed to identify the nature of the difficulties encountered, which referred to behaviors linked to certain executive functions. Overall, the results showed that children with ADHD seem to have more difficulties with routines in general and tend to experience more negative impacts than the two other groups of children. Moreover, children with ASD differed little from neurotypical children, which suggests that the difficulties experienced in carrying out routines are similar between these two groups. However, it is important to keep in mind that the autistic children who participated in this study were all able to use a digital application relatively independently, which may not be representative of all autistic children. Therefore, the absence of differences between neurotypical children and those with ASD could be due to a sampling bias.

Firstly, in terms of the most difficult routines to carry out, the results indicate that children with ADHD have significantly more difficulty with the morning routine or homework than neurotypical children or children with ASD. Similar to Firmin and Phillips' (51) qualitative study, children with ADHD found the morning routine to be the second most complex, primarily due to its multiple steps that require completion within a specific timeframe, such as leaving for school and work. However, according to the qualitative study of Schaaf et al. (32), this routine is also difficult to achieve for children with ASD, for the same reason mentioned above. The use of a different method for data collection may explain this discrepancy in results. In the present study, children with ASD did not differ from neurotypical children in regard to difficulties with the morning routine, suggesting that the difficulties encountered were similar. Secondly, compared to their neurotypical peers, children with ADHD experience the highest level of difficulty with the homework routine. This result converges with those obtained in the study by Firmin and Phillips (47), who stipulate that the after-school routine, particularly homework, is very difficult for children with ADHD. Indeed, many of the parents who took part in this study described the routine as “chaotic” and “stressful”. Furthermore, the current study found no significant differences in the challenges faced during the mealtime routine. However, Marquenie et al. (33) qualitative study of families of children with ASD reported that the mealtime routine was the most difficult to carry out because of the child's sensory particularities. Schaaf et al. (32) add that the sensory particularities of the autistic child often mean that parents have to cook several meal options. As a result, parents focus the vast majority of their attention on their child with ASD, to the detriment of other family members (32). However, the present study did not observe this specific difficulty among families of children with ASD. One possibility is that the participating families are already adapting the mealtime routine to their children's sensory needs, which may explain why this group doesn't seem more affected than the other two. Finally, we observed no significant difference in the difficulties encountered during the evening routine between the three groups of children. The predictability of the bedtime routine allows parents and children to spend more quality time together, which could explain this result (33).

In terms of the impact of routine performance on the child, results indicate that children with ADHD experience significantly more frustration and receive more reprimands compared to their neurotypical peers. According to Seymour et al. (60), children with ADHD have a much lower threshold for frustration than other children. These children are more likely to feel anger and express it when faced with difficulty. Also, since children with ADHD seem to have more difficulty than their peers in carrying out routines, their parents have to intervene, thereby increasing the number of reprimands they receive (55).

Considering the impact of routines on family life, several results are significant. Compared to parents of neurotypical children, the parents of children having ADHD claim that their child's difficulties have a major influence on their patience, the home dynamic, and their attendance at work. In the same perspective, Corcoran et al. (53) report that parents of children with ADHD have a more stressful family climate, notably because of the consequences of ADHD on various spheres of life. Indeed, the severity of ADHD symptoms can have a significant impact on the family environment, which in turn can increase parents' stress levels (61). Despite research identifying stable daily routines as a successful strategy for children with ADHD (47, 48), parents often find updating these routines challenging and stressful (14, 53). Furthermore, compared to neurotypical children, the current results reveal that the family climate is more unpleasant which aligns with studies showing that the climate in ADHD family is characterized by a higher recurrence of quarrels, repetition of instructions, and tensions (5, 62). Also, the challenges associated with routine with children having ADHD, particularly in executive functions, could impede or postpone the accomplishment of daily tasks, resulting in delays in parents' personal timetables. For instance, Corcoran et al. (53) highlighted the issue of lateness at work as a contributing factor. The present study's results suggest that parents of children with ADHD require increased verbal cues, repetition, and constant monitoring to ensure task completion. These findings are congruent with those of Shikerkar and Vajaratkar (5), who report that the experience of these parents is characterized by notable repetition of instructions and frequent monitoring of actions, as well as the need to support their child in a routine with many verbal cues, particularly about the steps and behaviours to be performed.

Lastly, we assessed the nature of the routine performance difficulties in all three groups of children. According to the results, children with ADHD had significantly more difficulties with behaviours related to self-regulation than children with ASD. One possible explanation for this difference is that children with ADHD may have difficulty performing an action despite environmental distractions, which impairs their concentration, according to some studies (44, 63). Secondly, the present study found no significant differences between the three groups in working memory-related behaviours, which differs from the results cited previously. Indeed, in children with ASD, there seems to be a greater prevalence of deficits in behaviours related to working memory, unlike other children (44, 64). However, in the review by Craig et al. (63), findings from several studies about difficulties associated with working memory between the three groups of children varied considerably. In addition, the present study found a significant difference between children with ADHD and neurotypical children regarding planning and time-management behaviours. However, the observed difference contrasts with the majority of studies reported in the review by Craig et al. (63), which suggested that children with ASD experience greater difficulties with time management than neurotypical children and even those with ADHD. Additionally, a small proportion of the included studies reported no significant group differences. The variability observed in the results can potentially be attributed to the different measuring instruments used to collect data on executive functions (e.g., questionnaires, cognitive tests, standardized tools). These instruments vary in precision and may conceptualize the functions differently. Finally, from a more global point of view, the results obtained from the RPQ indicate that children with ADHD have significantly more difficulty executing routines than their neurotypical peers. They also expose that children with ASD do not show more difficulties than neurotypical or ADHD children. This is an interesting result since the meta-analysis by Ceruti et al. (45) confirms that children with ADHD generally have more difficulty than their neurotypical peers with behaviors associated with routine performance, but Ceruti et al. (45) also states that children with ASD present greater difficulties in routines than neurotypical children. This diverges from the results obtained in the present study, since the nature of the difficulties of children with ASD do not differ from the other two groups in terms of routine performance. Beyond the interpretation of group differences in routine performance, these findings also raise important considerations regarding the measurement properties of the RPQ and the way group differences are captured across its subscales and total score. While children with ADHD showed significantly greater difficulty with behavioral self-regulation than children with ASD (*p* < .05), the total score only detected differences between neurotypical children and those with ADHD. This pattern is consistent with the multidimensional structure of the RPQ and contemporary models of executive functions that recognize their fractionated nature (34, 66, 67). It suggests that subscale analysis should be prioritized over exclusive reliance on the total score, particularly for intervention planning, as it allows identification of specific domains requiring support. The total score nonetheless retains utility as a global indicator for screening purposes.

In sum, it is important to note that the study sample consisted exclusively of French-speaking families from Quebec, a context characterized by specific cultural norms, sociolinguistic practices, and family routines. Cultural and societal values may have shaped parental expectations regarding child autonomy, involvement in daily activities, and the organization of routines, as well as parents' interpretations of what constitutes a “difficulty” in routine execution. In this regard, the discrepancies observed between the study's findings and the existing literature may reflect the unique cultural and sociolinguistic characteristics of the sample. Consequently, these socio-cultural factors should be considered when interpreting the results, and caution is warranted in generalizing the findings to families from other linguistic or cultural contexts.

### 4.1 Study limitations and future directions

There are a number of limitations to the study that need to be addressed. Firstly, among the sample of children who took part in the study, 50.7% had ADHD, 23.9% had ASD and 25.7% had no diagnosis. Since the three groups in the study are represented by unbalanced proportions, it is possible that differences between neurotypical children and children with ASD could not be detected due to a lack of statistical power. In this sense, it would be relevant to carry out a similar study with a more equitably distributed sample in terms of their sociodemographic characteristics including the diagnosis and age. Also, although the children's diagnosis was confirmed by the BASC-3, the subtype of attention deficit (inattention, hyperactivity, combined) was not recorded by the research team. This may have influenced the results obtained, since according to Craig et al. (63), the inattention-only subtype shows fewer deficits in executive functions. Thus, it might have been interesting to see the distribution of the 104 children with ADHD according to the predominant subtype. By making this distinction, it will be possible to discriminate differences in routines within the group of children with ADHD. Still relating to the sample, only the child's main diagnosis was considered in the analyses, although many had a dual diagnosis (e.g., anxiety disorder, Gilles de la Tourette, oppositional disorder). Considering the fact that many children with ASD or ADHD have comorbid disorders, taking this into account in a future study could potentially allow us to observe whether the presence of comorbidities exacerbates the difficulties encountered in performing routines. Furthermore, as mentioned above, the present study is part of a larger research project. It is therefore important to mention that the families who took part in the project wanted to improve the performance of routines with their child, which may have influenced the fact that neurotypical children present more difficulties than the average. Also, as the children had to be able to use a digital application during the project, it is possible that the children with ASD who took part presented a higher-level profile and thus do not represent the breadth of the child ASD population. These two elements, relative to the sample, may have had an impact on the differences observed between the groups, notably by having had a mitigating effect. This study presents an important conceptual limitation, as it relies predominantly on a deficit-based framework. The RPQ measures deviations from neurotypical routine execution patterns but does not distinguish between difficulties that genuinely impair functioning and neurodivergent approaches to routines that may be different yet functional. For example, an autistic child's strong preference for routine consistency or a child with ADHD's need for movement breaks during routines may represent adaptive strategies rather than deficits. Future research should incorporate a neurodiversity-informed approach by: (1) examining whether reported “difficulties” actually impact child well-being and family quality of life, (2) assessing families' own adaptive strategies and their effectiveness, and (3) potentially developing alternative scoring or interpretation guidelines that distinguish between dysfunctional patterns and neurodivergent but functional approaches. Finally, the use of a mixed descriptive-comparative research design could have provided a deeper understanding of the findings. The qualitative component would have complemented the quantitative analyses by offering a more detailed exploration of participants' experiences. This approach could have helped identify convergences and divergences between quantitative data and participants' narratives, thereby supporting, refining, or challenging certain conclusions. In a future study, it could also be interesting and relevant to include the perspectives of both caregivers regarding the child's difficulties and their impact on family functioning. By obtaining the views of two respondents on the same research subject, it becomes possible to triangulate the information, providing a more accurate picture of the situation. This approach also allows for examining whether the observed differences remain consistent regardless of which parental figure takes part in the research project.

### 4.2 Contribution of the article

This article contributes to the advancement of knowledge about the performance of daily routines in Quebec families in Canada. The results highlight difficulties more commonly encountered in families with a child having ADHD. From a clinical point of view, these results enable us to better understand the experiences of these families so that we can support them more appropriately. More specifically, the interventions put in place must take into account both the child's particularities and those relating to the environment in which he or she evolves [e.g., home, family composition; (61)]. Opting for interventions that meet the needs of all family members while taking into account how the family climate is affected by day-to-day difficulties will help improve routines, on the one hand, and increase positive interactions between parents and children, on the other. To this date, various efficacious intervention programs exist to support families in carrying out daily routines, including the Kairos serious game (65), which is proving useful for both children with ADHD, ASD, or neurotypical disorders and their parents.

## 5 Conclusion

The aim of this study was to compare neurotypical children, and children with ASD or ADHD in regard to the performance of daily routines. From the parents' point of view, several differences emerged as significant between the three groups of children. In general, it seems more difficult for children with ADHD to carry out routines, unlike the other two groups. As a result, the daily lives of these families showed to be more stressful and unpleasant. Also, the nature of the difficulties encountered differed somewhat between the three groups, showing that children with ADHD had more difficulty with behaviours linked to self-regulation, as well as with carrying out routines in general. Thus, according to the results obtained, routines must not only be adjusted according to the time of day, the time of year, and the child's age, as suggested by Barkley (30), but they must also be adapted according to each child's personal characteristics. Furthermore, effective interventions should build on the strengths and adaptive strategies that families already employ, recognizing that differences in routine execution may reflect functional, neurodivergent approaches rather than deficits. By prioritizing support for these existing strategies, interventions can foster meaningful, individualized outcomes instead of enforcing conformity to normative patterns. In sum, this study highlighted the significant differences experienced in the daily lives of the participating families.

## Data Availability

The datasets presented in this article are not readily available due to confidentiality considerations and in accordance with the research ethics board agreement and the participants' informed consent form. It is not possible to make requests to access the datasets. Further queries can be directed to the corresponding author.

## References

[1] Boyd BA McCarty CH Sethi C. Families of children with autism: a synthesis of family routines literature. J Occup Sci. (2014) 21(3):322–33. 10.1080/14427591.2014.908816

[2] Fiese BH. Routines and rituals: opportunities for participation in family health. OTJR Occup Particip Health. (2007) 27(1):41S–9. 10.1177/15394492070270S106

[3] Hosokawa R Tomozawa R Katsura T. Associations between family routines, family relationships, and children’s behavior. J Child Fam Stud. (2023) 32(12):3988–98. 10.1007/s10826-023-02687-w

[4] Romano M Lorio C Delehanty A Eugenio J Abarca D Trivedi B Family routines within caregiver-implemented early interventions: a scoping review. J Early Interv. (2022) 44(4):371–92. 10.1177/10538151211062206

[5] Shikerkar D Vajaratkar PV. Understanding daily routine and schedule of children with attention-deficit hyperactivity disorder: a qualitative study. Indian J Occup Ther. (2022) 54(3):96–101. 10.4103/ijoth.ijoth_26_21

[6] Crespo C Santos S Canavarro MC Kielpikowski M Pryor J Féres-Carneiro T. Family routines and rituals in the context of chronic conditions: a review. Int J Psychol. (2013) 48(5):729–46. 10.1080/00207594.2013.806811 23848452

[7] Bowling A Blaine RE Kaur R Davison KK. Shaping healthy habits in children with neurodevelopmental and mental health disorders: parent perceptions of barriers, facilitators and promising strategies. Int J Behav Nutr Phys Act. (2019) 16:1–10. 10.1186/s12966-019-0813-6 31242904 PMC6595579

[8] Dunn L Coster WJ Cohn ES Orsmond GI. Factors associated with participation of children with and without ADHD in household tasks. Phys Occup Ther Pediatr. (2009) 29(3):274–94. 10.1080/01942630903008327 19842856

[9] Liu Y Merritt DH. Family routines and child problem behaviors in fragile families: the role of social demographic and contextual factors. Child Youth Serv Rev. (2021) 129:106187. 10.1016/j.childyouth.2021.106187

[10] Moen ØL Hall-Lord ML Hedelin B. Living in a family with a child with attention deficit hyperactivity disorder: a phenomenographic study. J Clin Nurs. (2014) 23(21-22):3166–76. 10.1111/jocn.12559 25453121

[11] Schlebusch L Samuels AE Dada S. South African Families raising children with autism spectrum disorders: relationship between family routines, cognitive appraisal and family quality of life. J Intellect Disabil Res. (2016) 60(5):412–23. 10.1111/jir.12292 27120985

[12] Martin AJ Nejad H Colmar S Liem GAD. Adaptability: conceptual and empirical perspectives on responses to change, novelty and uncertainty. Aust J Guid Couns. (2012) 22(1):58–81. 10.1017/jgc.2012.8

[13] Mendes CG Mancini MC Miranda DM. Household task participation of children and adolescents with ADHD: a systematic review. Cad Bras Ter Ocup. (2018) 26(3):658–67. 10.4322/2526-8910.ctoAR1184

[14] Bagatell N. The routines and occupations of families with adolescents with autism spectrum disorders. Focus Autism Other Dev Disabil. (2016) 31(1):49–59. 10.1177/1088357615587503

[15] Bridley A Jordan SS. Child routines moderate daily hassles and children’s psychological adjustment. Children’s Health Care. (2012) 41(2):129–44. 10.1080/02739615.2012.657040

[16] Coussens M Van Driessen E De Baets S Van Regenmortel J Desoete A Oostra A Parents’ perspectives on participation of young children with attention deficit hyperactivity disorder, developmental coordination disorder, and/ or autism spectrum disorder: a systematic scoping review. Child Care Health Dev. (2020) 46(2):232–43. 10.1111/cch.12735 31867727

[17] Delemere E Dounavi K. Parent-implemented bedtime fading and positive routines for children with autism spectrum disorders. J Autism Dev Disord. (2018) 48:1002–19. 10.1007/s10803-017-3398-4 29177618 PMC5861169

[18] Harris AN Stoppelbein L Greening L Becker SP Luebbe A Fite P. Child routines and parental adjustment as correlates of internalizing and externalizing symptoms in children diagnosed with ADHD. Child Psychiatry Hum Dev. (2013) 45:243–53. 10.1007/s10578-013-0396-4 23868356

[19] Kitsaras G Goodwin M Allan J Pretty IA. Defining and measuring bedtime routines in families with young children: a delphi process for reaching wider consensus. PLoS One. (2021) 16(2):e0247490. 10.1371/journal.pone.0247490 33626107 PMC7904169

[20] McRae E Stoppelbein L O'Kelley S Fite P Smith S. Comorbid internalizing and externalizing symptoms among children with ADHD: the influence of parental distress, parenting practices, and child routines. Child Psychiatry Hum Dev. (2020) 51:813–26. 10.1007/s10578-020-01019-z 32607913

[21] Baker S Morawska A Mitchell A. Promoting children’s healthy habits through self-regulation via parenting. Clin Child Fam Psychol Rev. (2019) 22(1):52–62. 10.1007/s10567-019-00280-6 30725307

[22] Selman SB Dilworth-Bart JE. Routines and child development: a systematic review. J Family Theory Rev. (2023) 16(2):272–328. 10.1111/jftr.12549

[23] Wildenger LK McIntyre LL Fiese BH Eckert TL. Children’s daily routines during kindergarten transition. Early Child Educ J. (2008) 36(1):69–74. 10.1007/s10643-008-0255-2

[24] Kitsaras G Goodwin M Allan J Kelly MP Pretty IA. Bedtime routines child wellbeing and development. BMC Public Health. (2018) 18(1):386. 10.1186/s12889-018-5290-3 29562892 PMC5861615

[25] Spagnola AM Fiese BH. Family routines and rituals: a context for development in the lives of young children. Infants Young Child. (2007) 20:284–99. 10.1097/01.IYC.0000290352.32170.5a

[26] Jordan SS. Further validation of the child routines inventory (CRI): relationship to parenting practices, maternal distress, and child externalizing behavior (Proquest information and learning [doctoral thesis]). Baton Rouge: Louisiana State University and Agricultural and Mechanical College (2004).

[27] Massé L Verreault M Verret C. Mieux Vivre Avec le TDAH à la Maison [Living Better with ADHD at Home]. Montréal: Chenelière Éducation (2011).

[28] Bagby MS Dickie VA Baranek GT. How sensory experiences of children with and without autism affect family occupations. Am J Occup Ther. (2012) 66(1):78–86. 10.5014/ajot.2012.000604 22389942 PMC3324972

[29] Weisner TS. Well-being, chaos, and culture: sustaining a meaningful daily routine. In: Evans GW Wachs TD, editors. Chaos and its Influence on Children’s Development: An Ecological Perspective. Washington, DC: American Psychological Association (2010). p. 211–24.

[30] Barkley RA. Taking Charge of ADHD: The Complete, Authoritative Guide for Parents. New York: Guilford Press (2020).

[31] Hodgetts S Nicholas D Zwaigenbaum L. Home sweet home? Families’ experiences with aggression in children with autism spectrum disorders. Focus Autism Other Dev Disab. (2013) 28(3):166–74. 10.1177/1088357612472932

[32] Schaaf RC Toth-Cohen S Johnson SL Outten G Benevides TW. The everyday routines of families of children with autism. Autism. (2011) 15(3):373–89. 10.1177/1362361310386505 21430016

[33] Marquenie K Rodger S Mangohig K Cronin A. Dinnertime and bedtime routines and rituals in families with a young child with an autism spectrum disorder. Aust Occup Ther J. (2011) 58(3):145–54. 10.1111/j.1440-1630.2010.00896.x 21599679

[34] Barkley RA. Emotional dysregulation is a core component of AHDH. In: Barkley RA, editor. Attention-deficit Hyperactivity Disorder: A Handbook of Diagnosis and Treatment. 4th ed. New York: Guilford Press (2015). p. 81–115.

[35] Sinzig J Evers D Lehmkuhl G Vinzelberg I. Executive function and attention profiles in preschool and elementary school children with autism spectrum disorders or ADHD. Int J Dev Disabil. (2014) 60(3):144–54. 10.1179/2047387714Y.0000000040

[36] American Psychiatric Association. DSM-5-TR Diagnostic and Statistical Manual of Mental Disorders, Revised Text. 5th ed. Washington, DC: Elsevier Masson (2023).

[37] Public Health Expertise and Reference Group. (2019). Surveillance of attention deficit disorder with or without hyperactivity (ADHD) in Quebec. Available online at: https://www.inspq.qc.ca/en/node/16968 (Accessed October 15, 2024).

[38] Public Health Agency of Canada. (2022). Autism spectrum disorder: Highlights from the 2019 Canadian health survey on children and youth. Available online at: https://www.canada.ca/content/dam/phac-aspc/documents/services/publications/diseases-conditions/autism-spectrum-disorder-canadian-health-survey-children-youth-2019/autism-spectrum-disorder-canadian-health-survey-children-youth-2019.pdf (Accessed October 15, 2024).

[39] Usami M. Functional consequences of attention-deficit hyperactivity disorder on children and their families. Psychiatry Clin Neurosci. (2016) 70(8):303–17. 10.1111/pcn.12393 27061213

[40] Delis DC. Delis Rating of Executive Functions. Bloomington: Pearson (2012).

[41] Hughes DM Turkstra LS Wulfeck BB. Parent and self-ratings of executive function in adolescents with specific language impairment. Int J Lang Commun Disord. (2009) 44(6):901–16. 10.1080/13682820802425693 19105067

[42] Townes P Liu C Panesar P Devoe D Lee SY Taylor G Do ASD and ADHD have distinct executive function deficits? A systematic review and meta-analysis of direct comparison studies. J Atten Disord. (2023) 27(14):1571–82. 10.1177/10870547231190494 37565325 PMC10637091

[43] Liu C Townes P Panesar P Lee SY Devoe D Arnold P Executive function in ADHD and ASD: a scoping review. Rev J Autism Dev Disord. (2024):1–14. 10.1007/s40489-024-00444-3

[44] Corbett BA Constantine LJ Hendren R Rocke D Ozonoff S. Examining executive functioning in children with autism spectrum disorder, attention deficit hyperactivity disorder and typical development. Psychiatry Res. (2009) 166(2-3):210–22. 10.1016/j.psychres.2008.02.005 19285351 PMC2683039

[45] Ceruti C Mingozzi A Scionti N Marzocchi GM. Comparing executive functions in children and adolescents with autism and ADHD: a systematic review and meta-analysis. Children. (2024) 11(4):473. 10.3390/children11040473 38671689 PMC11049008

[46] Lanza HI Drabick DA. Family routine moderates the relation between child impulsivity and oppositional defiant disorder symptoms. J Abnorm Child Psychol. (2011) 39(1):83–94. 10.1007/s10802-010-9447-5 20690009 PMC3066087

[47] McRae EM Stoppelbein L O'Kelley SE Fite P Greening L. Predicting child behavior: a comparative analysis between autism spectrum disorder and attention deficit/hyperactivity disorder. J Child Fam Stud. (2019) 28:668–83. 10.1007/s10826-018-1299-6

[48] Stoppelbein L Biasini F Pennick M Greening L. Predicting internalizing and externalizing symptoms among children diagnosed with an autism spectrum disorder: the role of routines. J Child Fam Stud. (2016) 25:251–61. 10.1007/s10826-015-0218-3

[49] Laugesen B Lauritsen MB Jørgensen R Sørensen EE Rasmussen P Grønkjær M. Living with a child with attention deficit hyperactivity disorder: a systematic review. JBI Evid Impl. (2016) 14(4):150–65. 10.1097/XEB.0000000000000079 27058250

[50] Sallee FR. Early morning functioning in stimulant-treated children and adolescents with attention-deficit/hyperactivity disorder, and its impact on caregivers. J Child Adolesc Psychopharmacol. (2015) 25(7):558–65. 10.1089/cap.2014.0160 26151738 PMC4576958

[51] Firmin MW Phillips A. A qualitative study of families and children possessing diagnoses of ADHD. J Fam Issues. (2009) 30(9):1155–74. 10.1177/0192513X09333709

[52] Allik H Larsson JO Smedje H. Health-related quality of life in parents of school-age children with asperger syndrome or high-functioning autism. Health Qual Life Outcomes. (2006) 4(1):1–8. 10.1186/1477-7525-4-1 16393335 PMC1360061

[53] Corcoran J Schildt B Hochbrueckner R Abell J. Parents of children with attention deficit/hyperactivity disorder: a meta-synthesis, part I. Child Adolesc Soc Work J. (2017) 34:281–335. 10.1007/s10560-016-0465-1

[54] Larson E. A caregiving and autism: how does children’s propensity of routineization influence participation in family activities? Occup Ther J Res. (2006) 26(2):69–79. 10.1177/153944920602600205

[55] Johnston C Mash EJ. Families of children with attention-deficit/hyperactivity disorder: review and recommendations for future research. Clin Child Fam Psychol Rev. (2001) 4(3):183–207. 10.1023/a:1017592030434 11783738

[56] Edmonds WA Kennedy TD. An Applied Guide to Research Designs Quantitative, Qualitative, and Mixed Methods. 2nd ed. Thousand Oaks, CA: SAGE Publications, Incorporated (2016).

[57] Reynolds CR Kamphaus RW. BASC-3^CDN−F^ Système D’évaluation du Comportement de L’enfant—troisième édition—version Pour Francophones du Canada, Questionnaire des Parents (PRS-C) Enfant 6-11 ans, Feuille de Travail Pour la Notation Manuelle Chez L’enfant, Feuille de Saisie des Données [BASC-3^CDN−F^ Child Behavior Rating System—third Edition—canadian Francophone Version, Parent Questionnaire (PRS-C) Child 6-11 Years, Child Manual Scoring Worksheet, Data Entry Sheet]. Bloomington: NCS Pearson, Inc (2015).

[58] Simonato I Massé L Lussier-Desrochers D Lemieux A et Ruel, R. Développement et validation du questionnaire sur l’exécution des habitudes quotidiennes [development and validation of a questionnaire on the performance of routines]. Rev Québ Psychol. (2024) 44(3):36–57. 10.7202/1114899ar

[59] Rutherford A. Introducing ANOVA and ANCOVA: A GLM Approach. London: Sage (2001).

[60] Seymour KE Macatee R Chronis-Tuscano A. Frustration tolerance in youth with ADHD. J Atten Disord. (2016) 23(11):1229–39. 10.1177/1087054716653216 27282378 PMC6541529

[61] Muñoz-Silva A Lago-Urbano R Sanchez-Garcia M Carmona-Márquez J. Child/adolescent’s ADHD and parenting stress: the mediating role of family impact and conduct problems. Front Psychol. (2017) 8:2252. 10.3389/fpsyg.2017.02252 29312090 PMC5744077

[62] Craig F Savino R Fanizza I Lucarelli E Russo L Trabacca A. A systematic review of coping strategies in parents of children with attention deficit hyperactivity disorder (ADHD). Res Dev Disabil. (2020) 98:103571. 10.1016/j.ridd.2020.103571 31931455

[63] Craig F Margari F Legrottaglie AR Palumbi R De Giambattista C Margari L. A review of executive function deficits in autism spectrum disorder and attention-deficit/hyperactivity disorder. Neuropsychiatr Dis Treat. (2016) 2016(12):1191–202. 10.2147/NDT.S104620 PMC4869784 27274255

[64] Demetriou EA Lampit A Quintana DS Naismith SL Song YJ Pye JE Autism spectrum disorders: a meta-analysis of executive function. Mol Psychiatry. (2018) 23(5):1198–204. 10.1038/mp.2017.75 28439105 PMC5984099

[65] Lussier-Desrochers D Massé L Simonato I Lachapelle Y Godin-Tremblay V Lemieux A. Evaluation of the effect of a serious game on the performance of daily routines by autistic and ADHD children. Adv Neurodev Disord. (2023) 7(4):566–78. 10.1007/s41252-023-00319-4 PMC9896450 36777795

[66] Barkley RA. Executive functioning and self-regulation viewed as an extended phenotype: implication of the theory for ADHD and its treatment. In: Barkley DRA, editor. Attention-Deficit Hyperactivity Disorder: A Handbook of Diagnosis and Treatment. 4th ed. New York, NY: The Guilford Press (2015b). p. 405–34.

[67] Brown TE. A New Understanding of ADHD in Children and Adults: Executive Function Impairments. New York, NY: Routledge (2013).

